# Identification of Autophagy-Related LncRNA to Predict the Prognosis of Colorectal Cancer

**DOI:** 10.3389/fgene.2022.906900

**Published:** 2022-08-11

**Authors:** Ling Duan, Yang Xia, Chunmei Li, Ning Lan, Xiaoming Hou

**Affiliations:** ^1^ Department of Oncology, The First Hospital of Lanzhou University, Lanzhou, China; ^2^ The First School of Clinical Medicine, Lanzhou University, Lanzhou, China; ^3^ Department of Oncology, The First People’s Hospital of Lanzhou, Lanzhou, China

**Keywords:** colorectal adenocarcinoma, long non-coding RNA, autophagy, overall survival, prediction model

## Abstract

**Objective:** To establish a prediction model based on autophagy-related lncRNAs and investigate the functional enrichment of autophagy-related lncRNAs in colorectal cancer.

**Methods:** TCGA database was used to extract the transcriptome data and clinical features of colorectal cancer patients. HADb was used to obtain autophagy-related genes. Pearson correlation analysis was performed to identify autophagy-related lncRNAs. The autophagy-related lncRNAs with prognostic values were selected. Based on the selected lncRNAs, the risk score model and nomogram were constructed, respectively. Calibration curve, concordance index, and ROC curve were performed to evaluate the predictive efficacy of the prediction model. GSEA was performed to figure out the functional enrichment of autophagy-related lncRNAs.

**Results:** A total of 13413 lncRNAs and 938 autophagy-related genes were obtained. A total of 709 autophagy-related genes were identified in colon cancer tissues, and 11 autophagy-related lncRNAs (AL138756.1, LINC01063, CD27-AS1, LINC00957, EIF3J-DT, LINC02474, SNHG16, AC105219.1, AC068580.3, LINC02381, and LINC01011) were finally selected and set as prognosis-related lncRNAs. According to the risk score, patients were divided into the high-risk and low-risk groups, respectively. The survival K–M (Kaplan–Meier) curve showed the low-risk group exhibits better overall survival than the high-risk group. The AUCs under the ROC curves were 0.72, 0.814, and 0.83 at 1, 3, and 5 years, respectively. The C-index (concordance index) of the model was 0.814. The calibration curves at 1, 3, and 5 years showed the predicting values were consistent with the actual values. Functional enrichment analysis showed that autophagy-related lncRNAs were enriched in several pathways.

**Conclusions:** A total of 11 specific autophagy-related lncRNAs were identified to own prognostic value in colon cancer. The predicting model based on the lncRNAs and clinical features can effectively predict the OS. Furthermore, functional enrichment analysis showed that autophagy-related genes were enriched in various biological pathways.

## Introduction

Colorectal cancer (CRC) is considered to be one of the leading causes of cancer death, and with the increasing incidence of colorectal cancer, its degree of harm has received increasing attention (Carr, 2018). The epidemiological investigation showed that CRC has been set as the third most incident cancer and the second most common in mortality among malignancies (Siegel et al., 2020). A previous study showed that the 5-year survival rate of CRC patients was merely 10–50%, which strongly affects the living quality (Miller et al., 2019). The histopathological types of CRC include adenocarcinoma, squamous cell carcinoma, and mucinous carcinoma. Among them, adenocarcinoma is the most common type, which occupied about 95% of CRC patients (Barresi et al., 2019).

The previous predicting analysis showed that CRC was associated with various clinical and environmental features including alcohol abuse, smoking, and different regions and countries (Øines et al., 2017; Nana and Edward, 2019). And nowadays, the tumor-node-metastasis (TNM) stage is set as the most common tool to predict the risk of patients suffering from malignancies (Weiser, 2018). However, the TNM stage predicting tool showed limited effects in prognostic prediction. With the development of transcriptome technology, a large number of sequencing results have been used to evaluate and predict the prognosis of cancer patients (Wang et al., 2020). Previous studies showed that autophagy-related genes and long non-coding RNA (lncRNAs), immune-related genes, and lncRNAs own preferable prognostic predicting effect on CRC (Wiener et al., 2014; Tokunaga et al., 2020). However, few studies investigate the combined predicting effects of sequencing data and clinical features.

Autophagy, a programmed cellular process, mainly contributes to the degradation and recycling of damaged cellular organelles and macromolecules (Kimmelman and White, 2017; Li et al., 2020; Ashrafizadeh et al., 2022). Dysfunction of the autophagic process has been associated with the onset and development of many human chronic pathologies, such as cardiovascular, metabolic, and neurodegenerative diseases as well as cancer (Francesca et al., 2018). Therefore, a large number of researchers tried to determine the regulation mechanism of autophagy (Lin et al., 2021; Liu et al., 2021; Zhou et al., 2021). Autophagy plays an important role in maintaining homeostasis. The process is controlled by autophagy-related genes (Zhou, Dong, 2021) and autophagy plays an important role in maintaining the homeostasis of the body. Previous studies showed that the autophagy process was already verified to be involved in the progress of cancer (Francesca, Sadia, 2018). The decreased expression of autophagy-related genes was associated with the acceleration of the cancer progress (Burada et al., 2015). And based on the specific function of autophagy, autophagy can be set as a potential prognostic predicting strategy in different types of tumors. Previous studies showed that autophagy-related genes can be an effective way to predict the prognosis of CRC (Thongchot et al., 2018).

Long non-coding RNAs (lncRNAs) were defined as RNA links with more than 200 nucleotides. The main function of lncRNAs was set to regulate the gene expression and protein synthesis (Liu et al., 2019). Also, lncRNAs are associated with cell proliferation, differentiation, and microRNA regulation (Jin et al., 2019). Previous studies showed that lncRNA participated in the regulation of cell autophagy by regulating the function of autophagy-related genes and proteins (Ulitsky and Bartel, 2013; Ashrafizaveh et al., 2021; Mirzaei et al., 2021; Mirzaei et al., 2022). HAGLROS as a lncRNA with 699bp can inhibit the autophagy process by regulating the mTOR signaling pathway (Frankel et al., 2017).

Therefore, we performed this study aiming to establish a prognostic model based on autophagy-related lncRNAs and clinical features in CRC patients.

## Materials and Methods

### Patient Datasets

**FIGURE 1:**
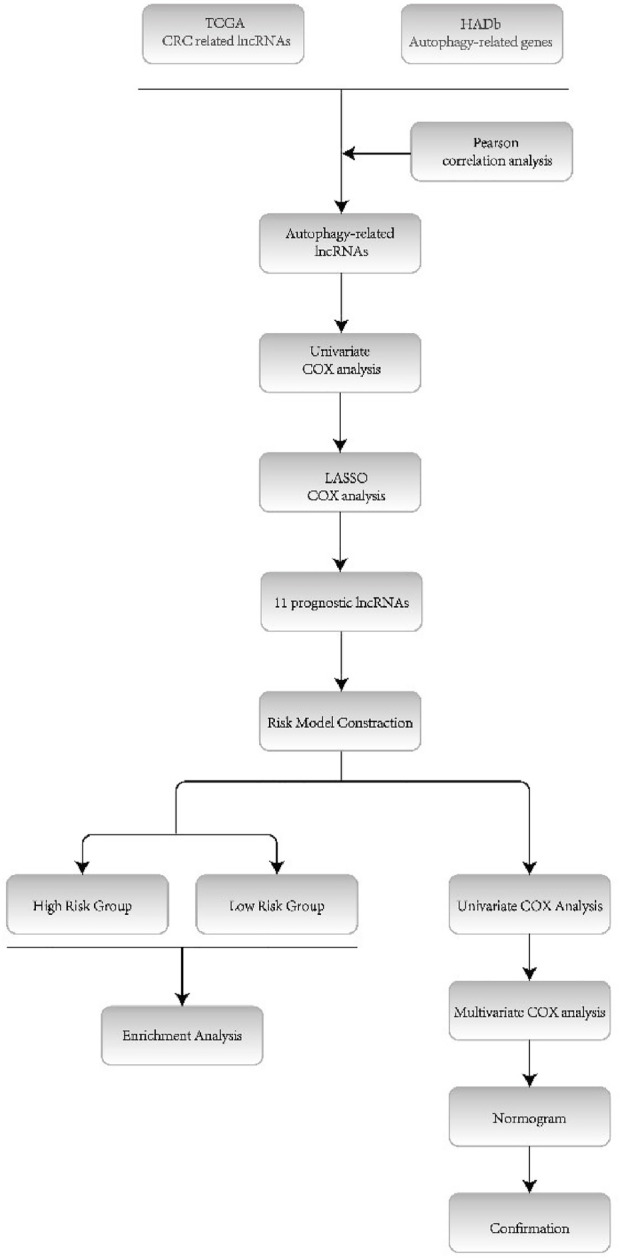
Flow diagram of this study.

Figure 1 illustrates the whole process of autophagy-related lncRNA CRC prediction model establishing. CRC datasets were downloaded from The Cancer Genome Atlas database (TCGA, https://cancergenome.nih.gov/), which includes clinical features, mRNA expression profiles, and lncRNA profiles. CRC patients whose overall survival (OS) time was less than 30 days or whose survival status was unknown were excluded from this study. Clinical features containing survival time, survival status, age, sex, grade, and TNM stage were included for further statistical analysis. All data were obtained from the open access TCGA database and therefore did not require the medical ethics committee approval.

Autophagy-related genes were downloaded from the Human Autophagy database (HADb, http://autophagy.lu/clustering/index. html). The “limma” package in R software was used to extract autophagy gene data from TCGA-CRC mRNA expression profiles. Pearson correlation analysis was performed to determine the autophagy-related lncRNAs. In this study, the correlation coefficient |R^2^| >0.3 and *p* < 0.001 (Wu et al., 2021) were defined as autophagy-related lncRNAs.

### Univariate COX Regression and Least Absolute Shrinkage and Selection Operator Regression Analysis

As Figure 1 shows, prognostic autophagy-related lncRNAs were confirmed by univariate COX regression analysis and absolute shrinkage and selection operator (LASSO) regression. Firstly, autophagy-related lncRNAs in univariate COX regression analysis whose *p* values were less than 0.05 were selected. Then, the selected lncRNAs were set into LASSO regression analysis.

### Multivariate COX Regression Analysis and Prognostic Model Establishment

All selected lncRNAs from LASSO regression were included in the multivariate COX regression model to generate their coefficient. Then, a prognosis prediction model was established with risk scores = ΣCoef * exp (genes). The risk score model was established based on coefficient values and expression levels. In this study, the median risk score was set as the cut-off value. And according to the cut-off value, patients were divided into high-risk groups and low-risk groups. The OS difference between groups was calculated by Kaplan-Meier (K-M) analysis.

Based on the clinical features and risk score of each colon cancer patient, we set univariate and multivariate COX regression analysis to construct the prognostic model. The nomogram was set to predict the 1-, 3-, and 5-years overall survival (OS) of each patient.

### Nomogram and Prognostic Model Evaluation

The predictive efficacy of the prediction model was evaluated by calculating the area under the ROC curve (AUC). And what is more, we also calculated the concordance index (C-index), a calibration curve to evaluate the predicted efficacy of the mixed model.

### Co-Expression Network and Gene Functional Enrichment Analysis

Based on the 11 prognosis-related lncRNAs identified, a co-expression network connecting these autophagy-related lncRNAs and autophagy-related genes was established. Protein-protein network (PPI) autophagy-related lncRNAs and their target genes were established based on Cytoscape 3.7.2 software (Cytoscape Consortium, San Diego, CA, United States). Gene Ontology (GO) enrichment analysis and Kyoto Encyclopedia of Genes and Genomes (KEGG) pathway analysis between the high-risk group and low-risk group was performed by Gene Set Enrichment Analysis (GSEA, http://www.broadinstitute. org/gsea/index.jsp). In total,1000 genome permutations were performed per analysis. The enrichment pathway screening conditions were: FDR< 0.25 and NOM *p* value <0.05.

### Statistical Analysis

All statistical analysis procedures were executed based on R (v4.0.3, New Zealand) software. Univariate cox analysis, LASSO regression, and multivariate cox analysis were used sequentially, and combined autophagy-related lncRNAs and clinical features to build prognostic models. Kaplan-Meier was performed to generate the survival curves. Log-rank analysis was utilized to compare the survival difference between the two groups. All statistical significances were set as *p* value less than 0.05.

## Results

### Differentially Expressed Autophagy-Related Gene and lncRNA Identification

A total of 417 CRC patients were screened from the TCGA database, the clinical baseline data of CRC patients were shown in Table1. Meanwhile, a total of 13413 lncRNAs were identified from the TCGA-COAD. A total of 938 autophagy-related genes were obtained from HADb. Finally, 709 autophagy-related genes were expressed in colon cancer tissues. Pearson correlation analysis showed that a total of 1342 autophagy-related lncRNAs were identified and selected.

**TABLE 1:** Clinical features of CRC patients from the TCGA database (*n* = 377).

		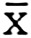 ±sd/n	%
Age (years)		59.32 ± 13.74	
Gender	Male	255	67.6
	Female	122	32.4
Stage	Stage I	175	46.4
	Stage II	87	23.1
	Stage III A	65	17.2
	Stage III B	9	2.4
	Stage III C	9	2.4
	Stage IV	2	0.5
	Stage IV A	1	0.3
	Stage IV B	2	0.5
	Unknow	24	6.4
T stage	Stage I	187	49.6
	Stage II	93	23.7
	Stage III	45	11.9
	Stage III A	29	7.7
	Stage III B	7	1.9
	Stage IV	13	3.4
M stage	M 0	272	72.1
	M 1	4	1.1
	M x	101	26.8
N stage	N 0	258	68.4
	N 1	4	1.1
	N x	115	30.5

### Univariate COX Regression and Least Absolute Shrinkage and Selection Operator Regression Analysis

**FIGURE 2:**
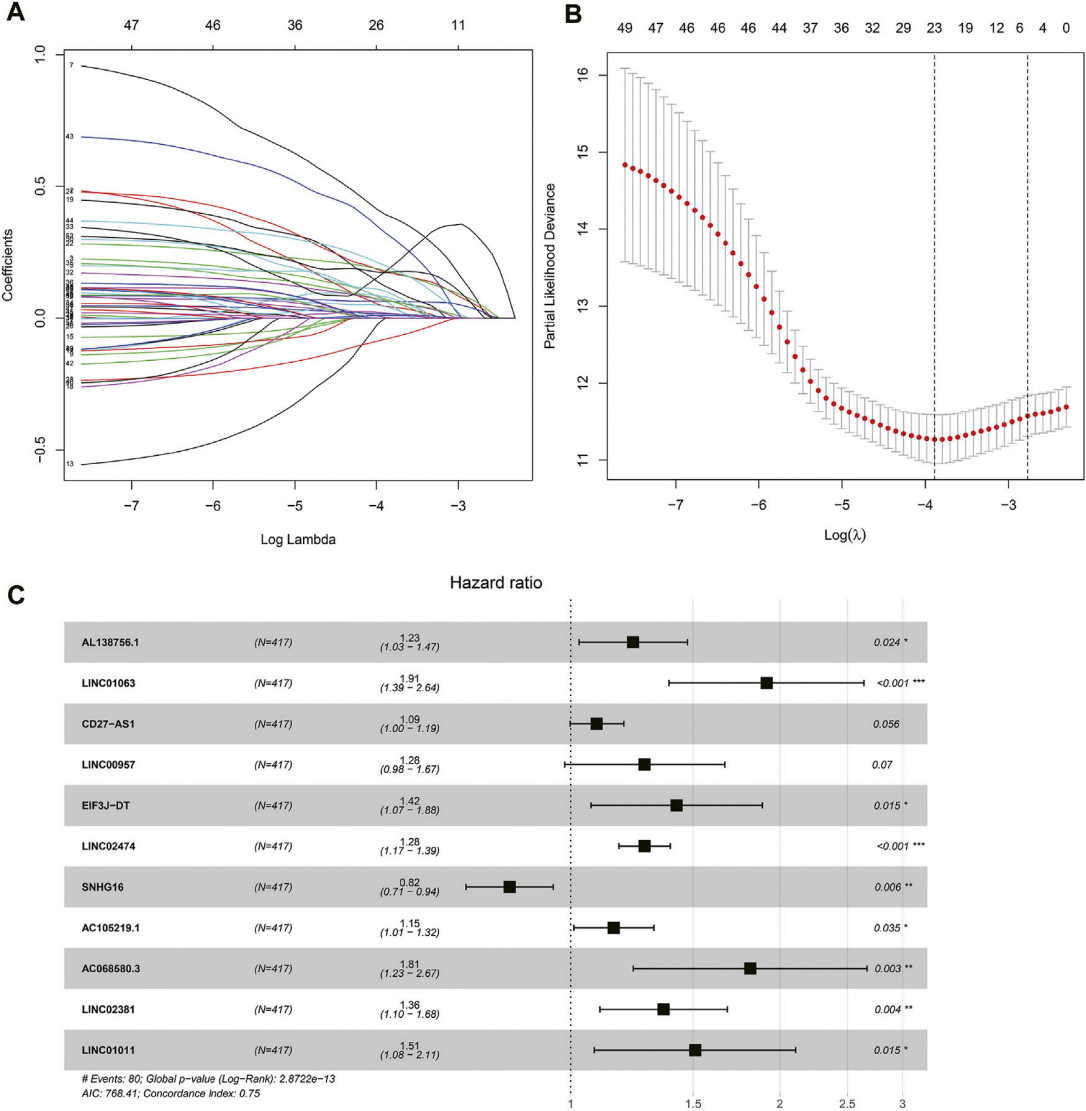
Autophagy related lncRNAs selection by COX regression and LASSO regression analysis. **(A)** The lasso regression model and cross validation method were used to screen autophagy-related lncNRAs. **(B)** Cross validation results. The regression coefficient map of genes in the LASSO model. **(C)** Cox proportional hazards regression analysis of 11 prognostic genes.

**FIGURE 3:**
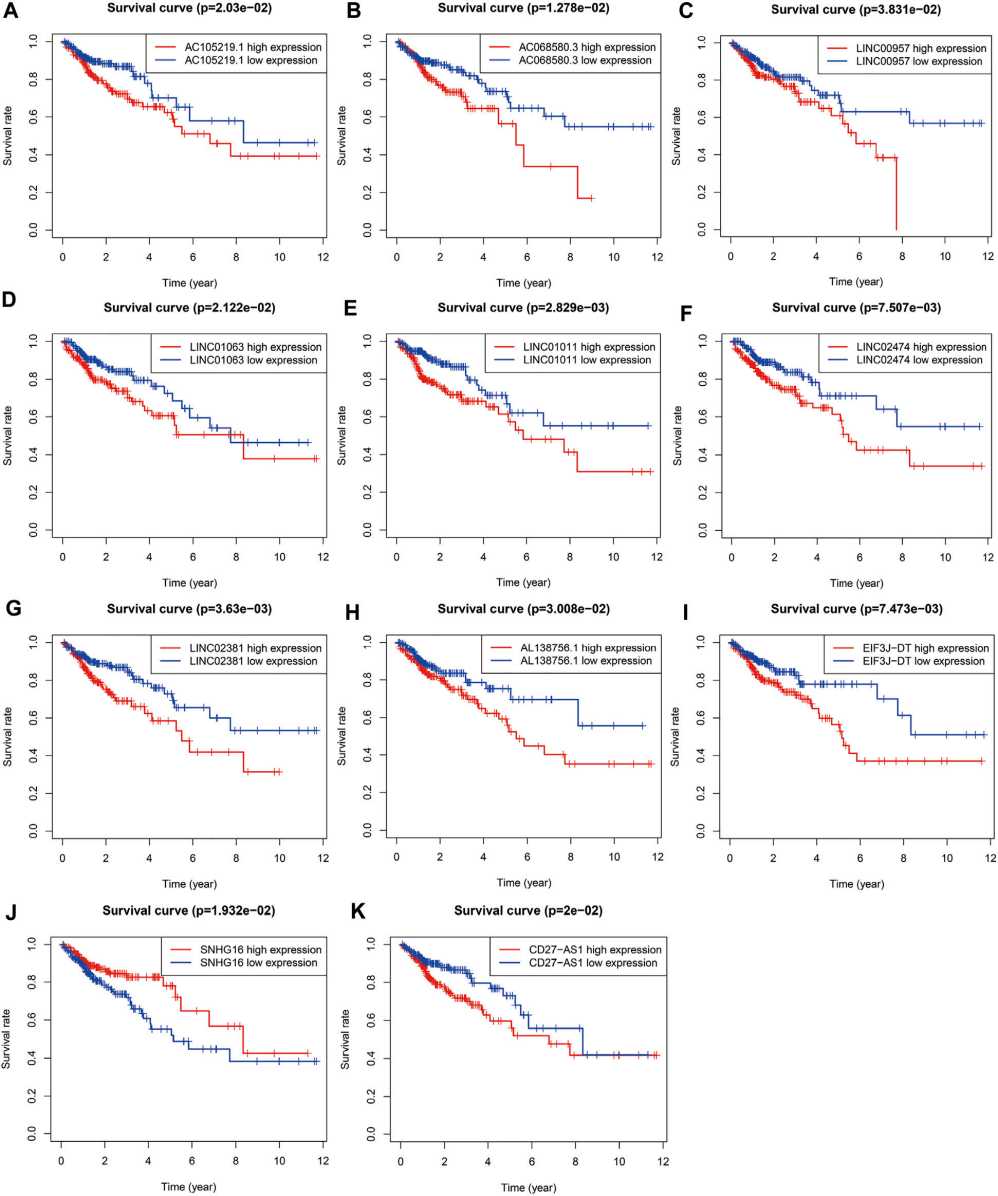
Survival curve of each autophagy related lncRNAs. Kaplan-Meier survival curves of 11 autophagy related lncRNAs were selected with *p* < 0.05 as the screening criteria. The red plots represent the high expression and the blue plots represent the median/low expression.

Univariate COX regression analysis selected 56 autophagy-related lncRNAs with *p* < 0.05. Eleven autophagy-related lncRNAs were finally selected by LASSO regression at the optimal values by using the 1 standard error (SE) of the minimum criteria (Figures 2A–C). K-M analysis based on 11 autophagy-related genes was shown in Figure 3.

### Risk Scoring Model Construction

**FIGURE 4:**
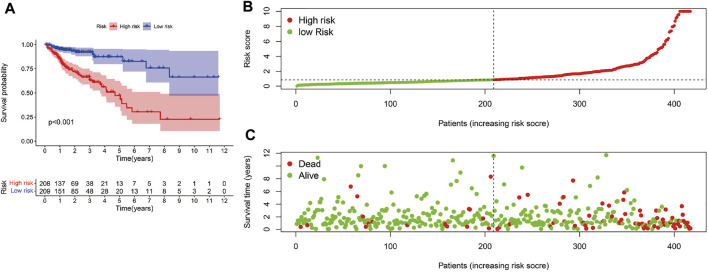
Risk score model evaluation. **(A)**The K-M curve c omparison between the high-risk group and low-risk group. **(B)** The Risk curve of high-risk and low-risk group. **(C)** Patients’ survival status distribution.

The risk scoring model was established by multivariate COX regression analysis. As we set the median risk score as the cut-off value, colon cancer patients included in our study were divided into high-risk group and low-risk group. The K-M survival curve showed that the difference of survival rate in the two groups was statistically significant (*p* < 0.01, Figure 4A). Meanwhile, the risk curve and scatter plot confirmed that compared with the high-risk group, the low-risk group had a lower risk factor and mortality, and the difference was significant (Figures 4B,C).

**FIGURE 5:**
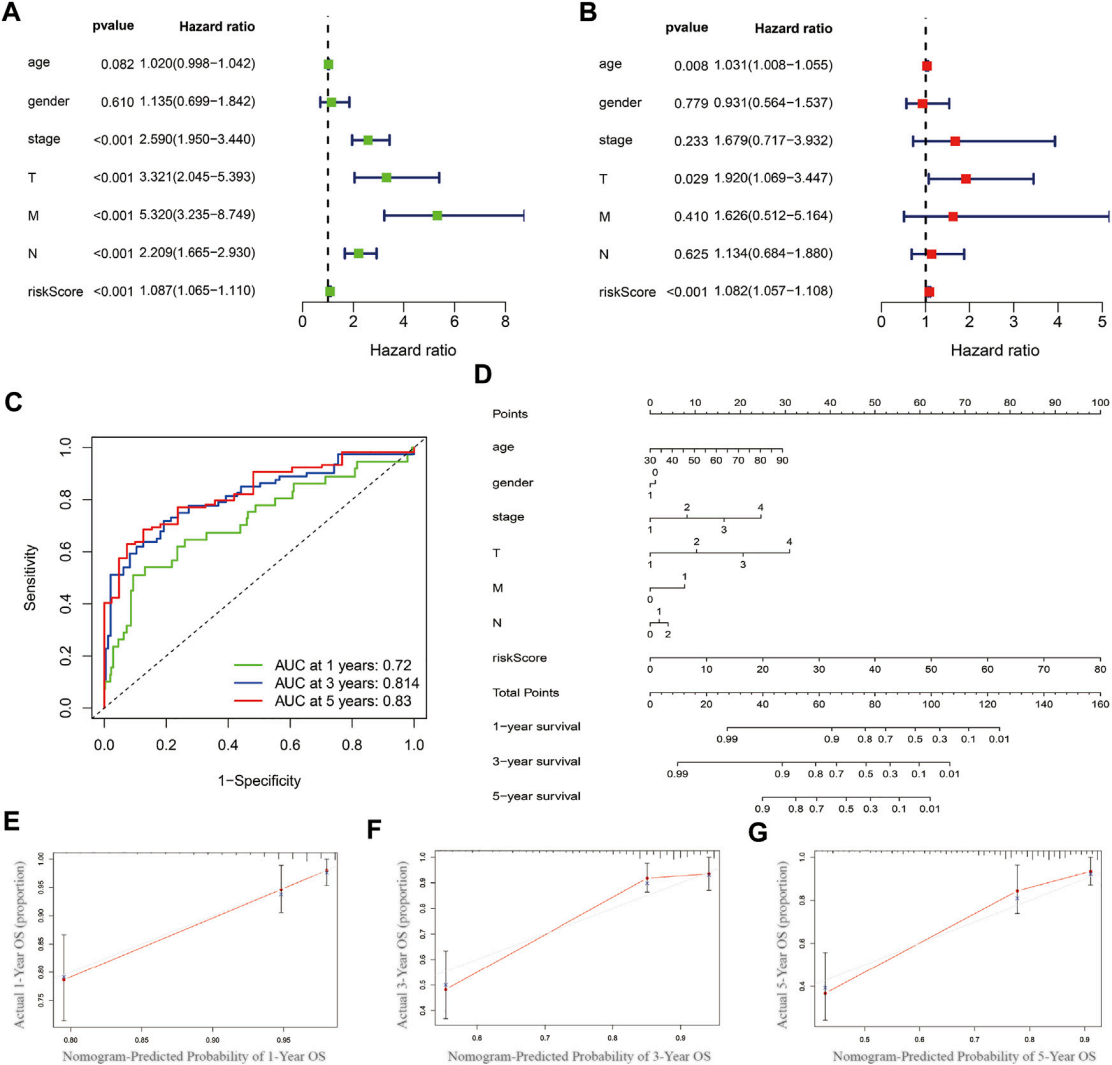
Prognostics predicting model establishing and validation. **(A)** Univariate COX regression analysis based on the clinical features and risk score. **(B)** Multivariate COX regression analysis. **(C)** Time-dependent ROC curve. **(D)** Nomogram based on multivariate COX regression analysis. **(E**–**G)** Calibration curve at 1-, 3- and 5-year.

The univariate and multivariate COX regression analysis based on the clinical features and risk score showed that the risk score was an independent prognostic factor of CRC patients (Figures 5A,B and Table 2). A time-dependent ROC curve was drawn and the AUCs were 0.72, 0.814, and 0.83 at 1-, 3-, and 5-years, respectively (Figure 5C).

**TABLE 2:** Multivariate COX regression analysis (*n* = 377).

Variable	*B*	*SE*	*Z*	*HR*	*HR*.95L	*HR*.95H	*p* value
Age	0.03	0.01	2.669	1.03	1.01	1.05	<0.01
Gender	−0.07	0.25	−0.28	0.93	0.56	1.53	0.77
Stage	0.51	0.43	1.19	1.67	0.71	3.93	0.23
T	0.65	0.29	2.18	1.91	1.06	3.44	0.02
M	0.48	0.58	0.82	1.62	0.51	5.16	0.41
N	0.12	0.25	0.48	1.13	0.68	1.87	0.62
Risk Score	0.07	0.01	6.65	1.08	1.05	1.11	<0.01

### Prognostic Model Establishing and Evaluation

**FIGURE 6:**
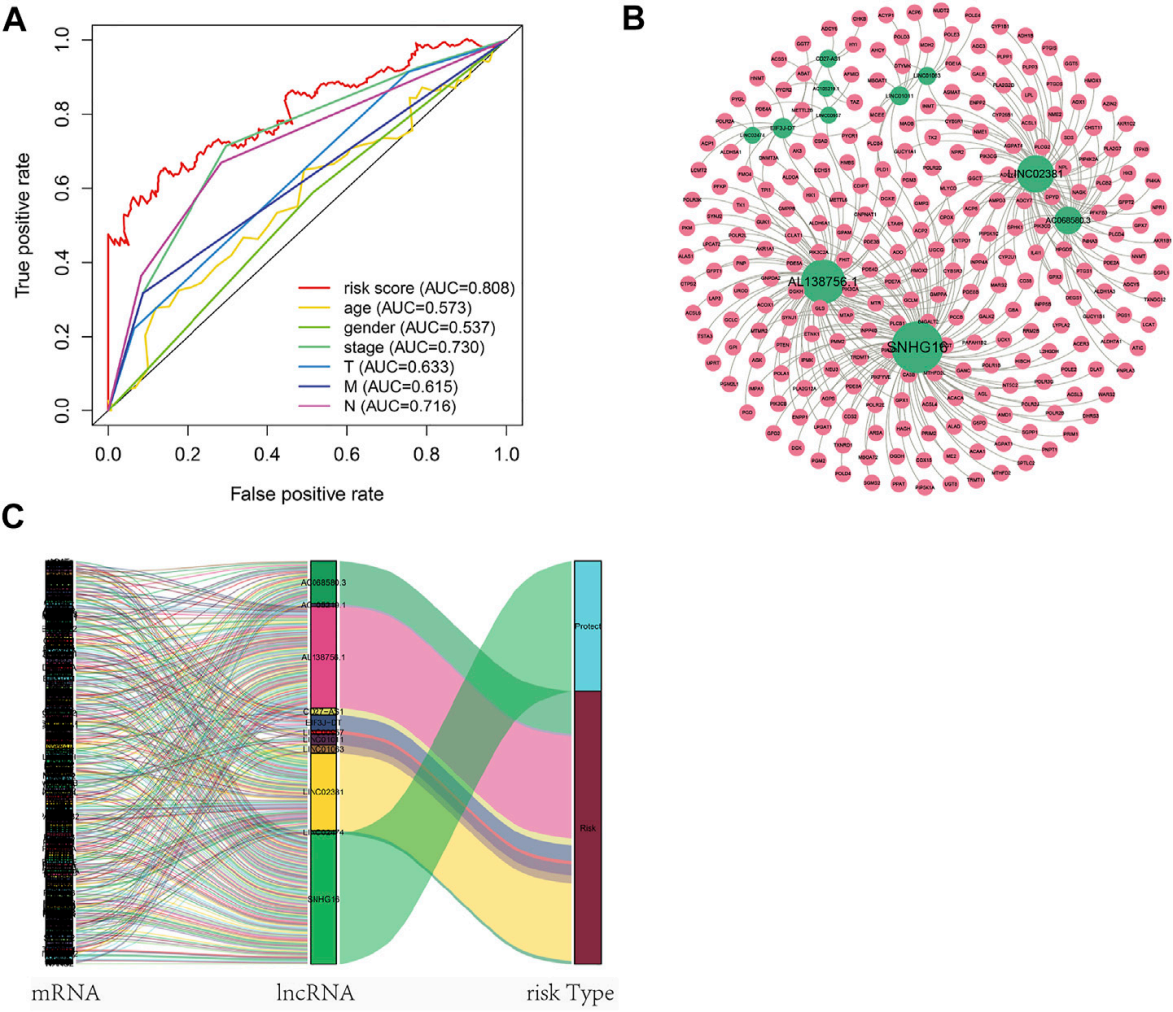
Autophagy-related lncNRAs and their target genes in CRC. **(A)** The ROC curve of each factor. **(B)** Network of autophagy-related lncNRAs and their target genes. **(C)** Sankey diagram of lncRNAs and linked genes.

Nomogram was established based on the risk score, age, gender, and TNM stages (Figure 5D). The results showed that 11 kinds of lincRNA may be the prognostic factors of colorectal cancer patients (all *p* < 0.05). Calibration curves at 1-, 3-, and 5-years were shown in Figures 5E–G. The AUCs of each factor at 5-year showed that the risk score, stage, and N stage showed certain prediction ability (0.808, 0.730, and 0.716, Figure 6A). A summary of gene ontology involving eleven lncRNAs (Table 3).

**TABLE 3:** Summary of gene ontology involving eleven lncRNAs.

lncRNAs	Gene Ontology	References
AL138756.1		Zhou et al. (2020)
LINC01063	GO_cytoplasmic_translational_initiation	
CD27-AS1	GO_ribonucleoprotein_complex_binding	
EIF3J-DT	GO_intramolecular_transferase_activity	
SNHG16	GO_ribosome_binding	
LINC02381	GO_nucleobase_biosynthetic process	
LINC01011		
LINC00957	--	--
LINC02474		
AC105219.1		
AC068580.3		

### Co-Expression Network and Gene Functional Enrichment Analysis

**FIGURE 7:**
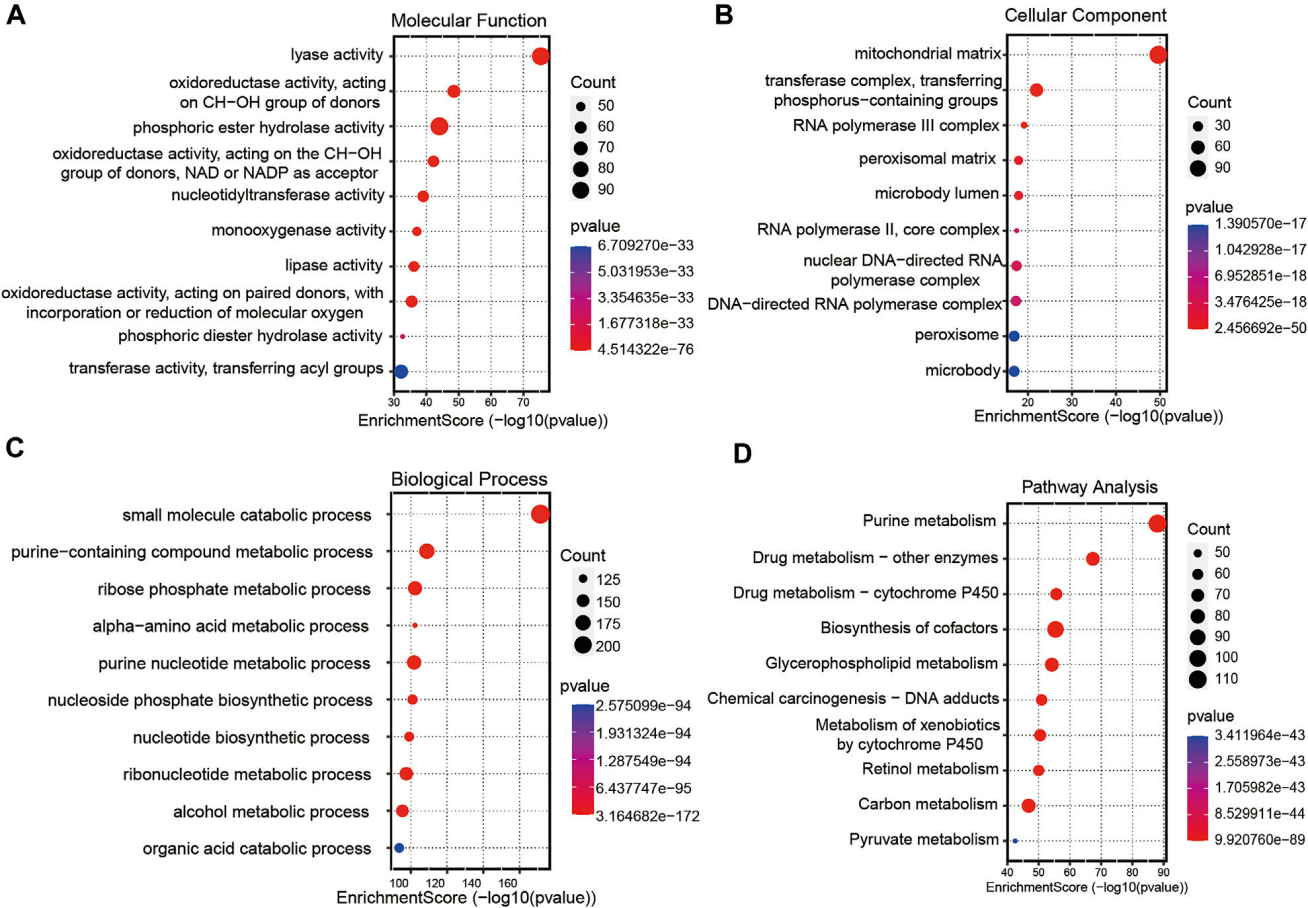
Gene functional enrichment analysis of autophagy-related lncNRAs. **(A)** The significant terms of MF (molecular function) enrichment. **(B)** The significant terms of CC (cellular component) enrichment. **(C)** The significant terms of BP (biological process) enrichment. **(D)** The top 10 significant terms of KEGG analysis.

A total of 256 autophagy-related genes were found to be associated with 11 prognosis-related lncRNAs. At the same time, we established a co-expression network based on the relationship between these genes and lncRNAs, with 267 nodes and 359 edges (Figure 6B). The Sankey diagram visually and clearly shows the association between each node and the patient’s prognosis (Figure 6C). Between the high-risk group and low-risk group, autophagy-related lncRNAs were enriched in GO terms: protein localization to the cilium, pseudouridine synthesis, tricarboxylic acid cycle, ciliary base, ciliary plasm, nucleoid, deacetylase activity, intramolecular transferase activity, NAD-dependent protein deacetylase activity, prenyltransferase activity, and KEGG terms: AXON guidance, citrate cycle TCA cycle, glycerophospholipid metabolism, glycosaminoglycan biosynthesis chondroitin sulfate, snare interactions in vesicular transport, terpenoid backbone biosynthesis, VEGF signaling pathway (Figures 7A–D).

## Discussion

The enhancement of autophagy can make CRC cells survive in the status of nutrition and energy deficiency. The enhanced autophagy also strengthened radiotherapy and chemotherapy resistance of CRC cells. And during biological therapy procedures, physicians tended to inhibit the cell autophagy to mediate the CRC cell programming death (Liu et al., 2018). Furthermore, previous studies have shown that cell autophagy was associated with the CRC prognosis (F, 2018). Based on these backgrounds, the autophagy-related lncRNA signature can be an effective and reliable indicator to predict the prognosis of CRC.

Until now, the specific mechanisms of autophagy in cancer progression have remained unclear, but substantial evidence suggests that autophagy has a great potential as a prognostic predictor in CRC (Lv et al., 2022; Zhu et al., 2022). We obtained the open access data from the TCGA database (TCGA-CRC) and comprehensively analyzed the association between autophagy-related lncRNAs and CRC by bioinformatics analysis. We aimed to screen for features that could be used to predict the CRC prognosis and guide treatment strategies, as these features may be new prognostic markers.

We extract autophagy-related lncRNAs from the TCGA database and HADb database by Pearson correlation analysis. We performed univariate cox analysis, LASSO regression, and multivariate cox, respectively, a total of 11 autophagy-related lncRNAs were finally selected: AL138756.1, LINC01063, CD27-AS1, LINC00957, EIF3J-DT, LINC02474, SNHG16, AC105219.1, AC068580.3, LINC02381, and LINC01011. Among the 11 autophagy-related lncRNAs, 8 lncRNAs were already verified to be associated with cancer development: LINC01063, CD27-AS1, LINC00957, EIF3J-DT, LINC02474, SNHG16, LINC02381, and LINC01011. Soudeh et al. compared breast cancer tissue with normal breast tissue and find that LINC01063 was significantly decreased in breast cancer tissue (Ghafouri-Fard et al., 2021). Anirban et al. concluded that down-regulated expression of CD27-AS1 was associated with the prognosis of cervical carcinoma (Ar et al., 2020). Zhang et al. reported that LINC00957 can be set as a prognostic marker in human CRC (Zhang et al., 2007). Luo et al. concluded that EIF3J-DT regulated the autophagy procedure in gastric cancer by targeting ATG14 (Luo et al., 2021). Du et al. found that LINC02474 mediated the CRC cell’s apoptosis by inhibiting the expression of GZMB (Du et al., 2021). Wu et al. concluded that SNHG16 facilitated the nasopharyngeal carcinoma progression by sponging miR-520a-3p to upregulate MAPK1 expression (Wu et al., 2021). Through the RT-PCR technique, Sun et al reported that LINC02381 enhanced CBX5 expression by binding the CBX5 promoter and activating CBX5 transcription in glioma (Sun et al., 2021). Song et al. reported that LINC01011 controlled mitochondrial fission by inhibiting BRCA1 transcription in squamous cell carcinoma (Fan et al., 2020). The remaining 3 lncRNAs (al138756.1, AC105219.1, and AC068580.3) have not been proved to be correlated with the progress and prognosis in CRC. There still needs further research on these lncRNAs.

Wu et al. had already confirmed that autophagy-related lncRNAs were associated with the poor prognosis of CRC patients (Wu, Yin, 2021). However, the AUCs of the model were 0.70, 0.76, and 0.68 at 1-, 3-, and 5-years, which meant that the predictive value of the model was at a moderate degree and needed to be improved. In our study, according to the risk score formula, patients were divided into the low-risk group and high-risk group by the median risk score. Similar to the previous studies (Fan, Tian, 2020; Yang et al., 2021), the K-M curve showed that low-risk group survival was longer than the high-risk group with significance (*p* < 0.05). The AUCs at 1-, 3-, and 5-years were 0.72, 0.814, and 0.83, which indicate that the predictive ability of the risk score signatured by autophagy-related lncRNAs were stable and the risk score can be set as an independent prognostic indicator. Based on the previous analysis, the nomogram was built by multivariate COX regression analysis and shown in Figure 5D. As shown in Figures 5A,B, the risk score occupied the largest concentration. C-index, AUCs, and calibration curve showed that the nomogram can be used as an effective tool to predict the prognosis of CRC patients. We were able to conclude that our prognostic model was valid and independent of other clinical factors such as TNM classification, clinical stage, age, and gender.

To further investigate the functions of prognostic autophagy-related lncRNAs, GO enrichment analysis and KEGG pathway analysis were performed between the high-risk group and low-risk group. As for GO terms, the lncRNAs were enriched in the protein localization to the cilium, pseudouridine synthesis, tricarboxylic acid cycle of biological process, ciliary base, ciliary plasm, nucleoid of cellular components and deacetylase activity, intramolecular transferase activity, NAD-dependent protein deacetylase activity, and prenyltransferase activity of molecular function. As for KEGG pathway analysis, lncRNAs were enriched in AXON guidance, citrate cycle TCA cycle, glycerophospholipid metabolism, glycosaminoglycan biosynthesis chondroitin sulfate, snare interactions in vesicular transport, terpenoid backbone biosynthesis, and VEGF signaling pathway. A previous study revealed that the AXON guidance genes were involved in cancer development (Li et al., 2009; Wei et al., 2020). EM et al. revealed that in CRC patients, AXON-related genes such as ROBO1 and ROBO2 were critical genes in cancer pathogenesis (Je et al., 2013). VEGF signaling pathway is tightly associated with tumor angiogenesis. Chao decreased the VEGF expression in CRC tumor tissues and found that tumor growth was significantly suppressed (Fang et al., 2021).

There are several limitations to this study. First, the study type was a retrospective cohort study, which may contain statistical bias. Second, as a retrospective study, the data derived from the public database lacks information including the treatment methods and recurrence time. Third, the sample size was relatively small which may influence the accuracy of the results. Finally, this study was just an analysis based on a public database. Further analyses *in vivo* and *in vitro* are needed to verify the conclusion.

## Conclusion

All in all, eleven specific autophagy-related lncRNAs were identified to own prognostic value in colon cancer. The predicting model based on lncRNAs and clinical features can effectively predict the OS.

## Data Availability

Publicly available datasets were analyzed in this study. This data can be found at: TCGA, https://cancergenome.nih.gov/.
